# Central Amino Acid Sensing in the Control of Feeding Behavior

**DOI:** 10.3389/fendo.2016.00148

**Published:** 2016-11-23

**Authors:** Nicholas Heeley, Clemence Blouet

**Affiliations:** ^1^Medical Research Council Metabolic Disease Unit, Institute of Metabolic Science, University of Cambridge, Cambridge, UK

**Keywords:** food intake, amino acids, protein, leucine, brain, appetite

## Abstract

Dietary protein quantity and quality greatly impact metabolic health *via* evolutionary-conserved mechanisms that ensure avoidance of amino acid imbalanced food sources, promote hyperphagia when dietary protein density is low, and conversely produce satiety when dietary protein density is high. Growing evidence supports the emerging concept of protein homeostasis in mammals, where protein intake is maintained within a tight range independently of energy intake to reach a target protein intake. The behavioral and neuroendocrine mechanisms underlying these adaptations are unclear. While peripheral factors are able to signal amino acid deficiency and abundance to the brain, the brain itself is exposed to and can detect changes in amino acid concentrations, and subsequently engages acute and chronic responses modulating feeding behavior and food preferences. In this review, we will examine the literature describing the mechanisms by which the brain senses changes in amino acids concentrations, and how these changes modulate feeding behavior.

## Introduction

Over the past 20 years, a large number of studies have refined our understanding of how neuroendocrine networks detect internal energy availability and modulate behavioral circuits controlling energy intake to maintain energy homeostasis (1). Food intake is also driven by factors independent of internal energy balance. This is well illustrated by the contribution of the sensory and hedonic value of a diet to the control of energy intake independently of energy homeostasis (2). In addition, the need for specific macronutrients or nutrients can affect appetite and food choices, but the mechanisms underlying how individual macronutrients influence feeding behavior or how appetite for specific macronutrients/nutrients influences energy intake remain unclear.

Ensuring sufficient consumption of protein is essential for growth, reproduction, and species survival (3). Animals, from insects to mammals, have evolved mechanisms to ensure quantitatively and qualitatively adequate protein intake (3, 4). Detection of lack or abundance of single amino acids can have profound acute and chronic effects on feeding behavior and food preference (5, 6). In addition, within a certain range, dietary protein content is a determinant of total energy intake (7). Moderately low-protein diets are associated with an increase in energy intake, adjusted to match minimum requirements for nitrogen and essential amino acids (EAA) (8). Conversely, high-protein diets reduce energy intake, presumably to prevent excessive amino acid levels potentially toxic for the brain (9). This remarkable bidirectional adjustment of energy intake based on dietary protein content has been proposed to target a protein intake of 15% across multiple species from insects to humans (10) and supports the idea that protein intake is regulated by homeostatic mechanisms somewhat independent of energy intake or intake of carbohydrate and fat.

In this review, we will examine the literature exploring how the brain monitors internal amino acid availability and how this central detection modulates food intake. We will not discuss in detail the peripheral mechanisms by which amino acids are sensed and how these mechanisms may interact with the brain to control food intake (8, 11).

## Diet-Induced Changes in Brain Amino Acid Concentrations

The unique morphological and functional properties of mammalian cerebral endothelial cells that form the blood–brain barrier (BBB) allow the brain to be protected from toxins and sheltered from variations in blood composition, presumably providing the central nervous system (CNS) with an optimal chemical environment for cerebral functions. Amino acid homeostasis is particularly critical in the brain, as a number of non-EAA – l-glutamate, l-aspartate, l-cysteine, l-homocysteine, glycine, alanine, and taurine – can act directly as neurotransmitters when released at the synapse, while other amino acids, l-tyrosine and l-tryptophan, serve as precursors for neurotransmitters, the catecholamines, and serotonin, respectively. In addition, branched-chain amino acids (leucine, isoleucine, and valine – BCAA) serve as precursors for the neurotransmitter glutamate and pathologically high BCAA concentrations, as seen in Maple Syrup Disease, cause excessive glutamatergic signaling and neurological symptoms (9).

Four facilitative saturable amino acid carriers have currently been identified to be expressed on the luminal side (blood side) of the mammalian BBB, maintaining intra-cerebral levels of amino acids within a narrow range to about 10% of plasma levels (12–14). However, the idea that all amino acids are non-specifically buffered to that fraction is challenged by discoveries demonstrating selective transport of amino acids across the BBB. This was first suggested by the observation that following arterial delivery of radiolabeled amino acids in rats, brain uptake of essential neutral amino acids is 5- to 10-fold greater than that of non-EAA (15, 16). Molecular support for this observation indicates that the system L1 amino acid transporter, which carries most EAA including branched-chain (leucine, isoleucine, and valine – BCAA) and aromatic (phenylalanine, tyrosine, and tryptophan – AAA) amino acids, is the predominant amino acid transport system expressed in the brain endothelium (13, 17, 18). In addition, the luminal and abluminal (brain side) membranes of the brain endothelium are functionally distinct, as sodium-dependent amino acid transport systems are present exclusively on the abluminal membrane, providing the BBB with a mechanism to actively export amino acids against the concentration gradient (13, 19). Thus, the BBB expresses transport systems that allow selective import and exports of amino acids and active regulation of brain extracellular amino acid composition.

BCAA and AAA compete for transport through system L1, and consequently, the blood ratio of BCAA to AAA levels is a major determinant of brain extracellular and cerebrospinal amino acid composition (9). Decreased plasma BCAA levels, as seen in specific contexts including endurance training or adaptation to ketogenic diets, are associated with decreased brain BCAA levels, increased uptake of tryptophan and tyrosine into brain, and increased synthesis of serotonin and catecholamines (20, 21). Conversely, increased plasma BCAA levels are reflected in brain BCAA levels, and negatively impact brain AAA uptake and serotonin synthesis (9, 13). Such changes have been reported in contexts associated with chronic increases in BCAA levels including diabetes or maintenance on a high-protein diet (12, 22–25) but also acutely following food ingestion (26). In fact, while the plasma level of most amino acids remains relatively stable in the postprandial period, BCAA levels rapidly and transiently rise as they largely escape first-pass splanchnic metabolism (27–29), resulting in a rapid increase in brain BCAA levels (26, 30–32). This observation led to the hypothesis that circulating BCAA levels may represent a signal of postprandial protein availability that regulates various anabolic functions modulated by dietary proteins. Consistently, BCAA have been shown to mediate protein-induced transcription, insulin secretion, and protein synthesis (33–35). Likewise, brain BCAA levels may mediate protein-induced modulations of centrally controlled functions, including appetite and metabolism, as discussed below.

Only a few studies have explored the regional differences in the kinetics of brain amino acid uptake following meal ingestion. Microdialysis studies in rats demonstrated that after oral gavage of a balanced amino acid mix or ingestion of a 50% protein meal, there were consistent increases in the concentrations of BCAA in the lateral and periventricular nuclei of the hypothalamus within 20–40 min of meal consumption, while the concentrations of the majority of other amino acids remained unchanged, with the exception of methionine and tyrosine that consistently increase in these brain regions following a meal (36–38). Whether this effect is specific to these brain regions or also occurs in other areas of the brain, including other hypothalamic nuclei, remains to be determined. In contrast, regional differences in the concentration of BCAA and AAA following the ingestion of amino acid imbalanced diets have been described. In this context, while the concentration of the limiting amino acid decreases in discrete sites, including the pyriform cortex, locus coeruleus, and the nucleus of the solitary tract, hypothalamic areas are protected from this deficiency (39–41). Collectively, these data indicate that amino acid concentrations in the brain are not a simple reflection of the plasma amino acid profile but vary selectively in discrete sites under specific dietary contexts.

## Central Detection of Essential Amino Acid Devoid or Unbalanced Diets

The marked reduction in energy intake and growth of animals maintained on diets containing very low protein amounts (<8% in rats; <5% in mice) or imbalanced EAA ratios was first described over 100 years ago (42). Seminal studies from Harper and colleagues demonstrated that the anorectic response to imbalanced amino acid diets is the cause rather than the consequence of growth failure, supporting a direct role for dietary amino acids in the regulation of food intake (43–46). Analysis of the behavioral responses to the ingestion of a diet devoid in one EAA indicated that the initial rapid anorectic response is followed by the onset of a learned conditioned taste aversion and the development of a specific appetite for the limiting amino acid (5, 47–51). These two latter adaptations have been associated with chronic changes in feeding-regulating circuits and are secondary to acute neuronal amino acid sensing, as reviewed in (52).

The rapid initial aversive response to EAA-devoid diets, manifested by a decrease in meal size and an increase in inter-meal interval, occurs within 20–40 min following feeding onset and is dependent on acute amino acid interoception. This response is independent of food sensory stimuli or peripheral signals (53–55) and instead relies on direct neuronal sensing of EAA imbalance by the anterior piriform cortex (APC). This assertion is supported by the following observations: (i) APC lesions prevent rats discriminating between AA containing and AA devoid diets (56, 57), (ii) concentrations of the limiting EAA in the APC rapidly fall after the introduction of the devoid diet (41, 48), through competition at the capillary endothelial amino acid transport system (58–60), and (iii) replacement of the limiting EAA into the APC *via* microinjections rapidly increases intake of a diet deficient in that EAA (49, 57, 61, 62). Importantly, this aversive response was shown to be independent of diet palatability and novelty (63). Thus, the APC is both necessary and sufficient to produce rapid hypophagia in response to EAA imbalanced diets.

**Figure 1:**
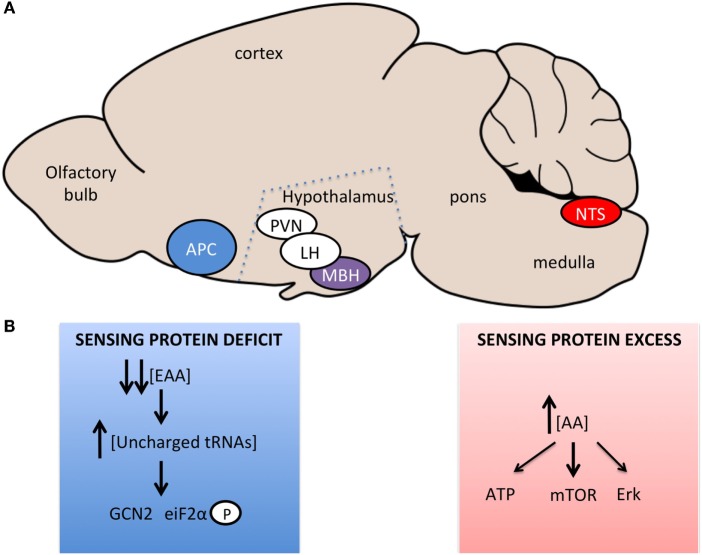
**Brain regions and signaling pathways involved in central amino acid sensing**. **(A)** Sagittal representation of the rodent brain with amino acid sensing brain regions identified. Blue – regions sensing amino acid imbalanced diets or very low-protein diets, Red – regions sensing increases in amino acid concentrations, Purple – regions involved in sensing bidirectional changes in amino acid concentrations, White – regions involved in neurocircuitory of amino acid sensing, which are not primary sensing sites. **(B)** Signaling pathways implicated in central sensing of amino acid deficit or excess.

Neurophysiological and neuroanatomical evidence further indicate that local APC EAA sensing initiates the response to EAA-devoid diets and engages neurocircuits connected to hypothalamic, pontine, and hindbrain feeding-regulating networks (Figure 1A). Neurons in the APC have been shown to be excited by the absence of threonine or the presence of the amino alcohols, which cause tRNA uncharging (61, 64). Changes in local interneuron interactions, at least in part *via* decreased local inhibitory GABAergic tone (65), cause a change in output signal from glutamatergic APC neurons (66) – their excitatory output is potentiated when GABAergic inhibitory control is lost. Tracing studies from the APC identified the projection targets of APC EAA sensing neurons [reviewed in Ref. (52)], but the functional relevance of the targets in the acute aversion to EAA deficiency has not been directly addressed. Two hypothalamic regions have been implicated in this acute response: the VMH and the LH, both rapidly activated in response to a lysine-deficient meal according to fMRI assessments in rats (67). Norepinephrine and dopamine levels are rapidly increased in these regions under these conditions (39, 62), providing some neurochemical insights into the circuits engaged from APC EAA imbalance chemodetection. Clearly, the precise circuits engaged downstream from the APC in mammals to produce the rapid aversion to EAA imbalance remain partially characterized, and novel circuit mapping tools would prove useful to decipher these circuits.

Work by two independent groups demonstrated that the rapid detection of dietary EAA deficiency within the APC occurs *via* a GCN2-dependent mechanism in mice; this pathway is also required for rejection of for EAA imbalanced diets in drosophila (68). The GCN2 pathway is an evolutionarily conserved pathway identified in yeast to mediate the detection of amino acid deficiency (69, 70). When cellular amino acid levels fall, uncharged tRNAs accumulate in the cell, bind to GCN2 that displays kinase activity toward eiF2α (eukaryotic initiation factor 2α), causing a global suppression of translation, but increased transcription of starvation relevant transcripts (Figure 1B). Evidence supporting a role for this pathways in the aversion to unbalanced EAA diet was obtained using GCN2 knockout mice, in which the rapid aversion to unbalanced diets is markedly blunted (71, 72) and downstream signaling, increased eiF2α phosphorylation (73), is absent (71, 72). In addition, direct injection of amino alcohols (that cause tRNA uncharging and GCN2 activation), into the APC of rats fed a normal diet, caused a suppression of feeding but had no effect when mice were on a diet devoid of the amino acid for which the matched amino alcohol was injected (71). This effect is specific to EAA, with proline and serine amino alcohols having no effect on feeding.

However, a recent study challenged these findings and failed to observe a rapid GCN2-dependent hypophagic response to threonine and leucine deficient diets (74, 75). Mice switched from a control to a leucine or threonine-devoid diet did not display a rapid hypophagic response during the first 3 h of feeding the novel diet but did show a hypophagic response after this time point. This delayed hypophagia was GCN2 independent. This latter observation is not necessarily in opposition with previous reports, as GCN2 signaling in the APC is restored within 2 h following the ingestion of an imbalanced diet and is not involved in the longer term hypophagic response to EAA imbalanced diets (76). However, the lack of acute aversion to the imbalanced diets in the Leib study contrasts with the rest of the literature. These discrepancies could be explained by diverging experimental paradigms (duration of fast, amino acid composition of the baseline diet) that may affect the production or kinetics of central EAA imbalance (77). In paradigms evidencing a rapid aversive response to EAA imbalance diets, a rapid fall in the APC concentration of the limiting amino acid occurred in the same time course as the production of the hypophagic response, within 40 min of diet exposure (41, 48, 78), and correlated with the activation of the GCN2 pathway in the APC (71, 72). In the Leib study, plasma concentrations of the missing amino acid and APC GCN2 signaling 1 h after diet exposure were unchanged, suggesting that the EAA imbalance failed to reach the APC during early exposure to the diet in these conditions.

More recently, the mediobasal hypothalamus has been proposed to be a primary sensing site of EAA deficiency (79). After an overnight fast, a leucine devoid diet caused an increase in eIF2α phosphorylation in the MBH within 40 min of diet consumption. Adenoviral-mediated knockdown of GCN2 in the MBH blunted the anorectic response to a leucine deficient diet over an hour of diet consumption. In addition, icv L – leucinol injection increased eiF2α phosphorylation selectively in the MBH and was sufficient to inhibit feeding in WT mice, but not GCN2 KO mice. Importantly, L – leucinol did not activate eiF2α signaling in the APC, demonstrating (i) the specificity of the protocol to target the MBH alone and (ii) the sufficiency of the MBH to initiate an aversive response to a leucine deficient diet. However, in earlier studies, no changes in the concentration of the limiting amino acid were found in three hypothalamic nuclei studied (39), suggesting changes in amino acid concentrations are not uniform across the brain. While the hypothalamus is clearly involved downstream of the APC in the anorectic response to AA deficient diets (80–82), further work will be needed to explore if the MBH is required for primary sensing of EAA deficiency.

While there is controversy about the mechanism and site(s) of central amino acid sensing in this context (75, 77), the ability of central amino acid absence to regulate feeding behavior is clear, and further work will be required to clarify these mechanisms and the neural circuits involved.

## Central Sensing of Amino Acid Abundance and the Control of Feeding Behavior

Early work from the Mayer and Harper labs identified that dietary supplementation with amino acids, in particular l-leucine, induces a hypophagic response comparable to that seen following adaptation to high-protein diets in rats (83, 84). Panskeep and Booth suggested that hypothalamic amino acid sensing may contribute to this anorectic response and reported that direct administration of a balanced mixture of amino acids into the hypothalamic parenchyma reduces food intake within 1 h following the injection (85). Subsequently, several groups confirmed that administration of physiologically relevant amounts of leucine into the 3rd ventricle or discrete brain nutrient-sensing regions of fasted rodents reduces energy intake during the subsequent refeeding period (26, 86–88). This anorectic response is not produced by other branched-chain amino acids or any aromatic amino acids (26, 87), is not accompanied by the development of conditioned taste aversion (26, 86), persists for 24 h, and produces a significant decrease in body weight gain (26, 86), as summarized in Table 1. Brain leucine levels are increased following a meal, and brain leucine administration reduces food intake, suggesting that brain amino acid levels may constitute a signal of energy and/or protein availability detected by brain nutrient-sensing regions that modulate homeostatic feeding-regulatory circuits.

**Table 1:** **Effects of centrally administered amino acids on food intake**.

Paper	Species	Conditions	Time of injection	Route	Amino Acid	Dose	Food intake
(86)	Rat	24 h fast	Before onset of dark cycle	3rd icv	Leucine	1.1 μg	↓
(88)	Rat	24 h fast	–	3rd icv	RPMI Amino Acid Mix	1 μg Leucine, and other EAA	↓
					Leucine	1, 3, 10 μg	↓
(89)	Rat	6 h fast	5–6 pm (onset of dark cycle)	3rd icv	Leucine	197 ng, 787 ng, 1.6 μg	No effect of 197 ng, others lowered feeding
(26)	Rat	OVN fast	1 h before onset of dark cycle	MBH	Leucine	56 ng	↓
	Mouse	6 h fast	1 h before onset of dark cycle	MBH	Leucine	28 ng	↓
					Valine	28 ng	No effect
(90)	Rat	OVN fast	1 h before onset of dark cycle	NTS	Leucine	28 ng	↓
					Valine	28 ng	No effect
(87, 91)	Rat	24 h fast	2 h prior to lights off	3rd icv	Leucine	10 ug	↓
	Rat	24 h fast	2 h prior to lights off	3rd icv	Tryptophan, Methionine, Lysine, Threonine, Serine	10 ug	No effect
	Rat	No fast LP diet	2 h prior to lights off	3rd icv	Leucine	10 μg	↓
(92)	Mouse	OVN fast	After overnight fast	3rd icv	Leucine	2.2 μg	No effect
(93)	Mouse	6 h fast	1 h before onset of dark cycle	NTS	Leucine	14 ng	↓

Leucine abundance is detected in the brain through neurochemically diverse and neuroanatomically distributed networks modulating feeding behavior. This is supported by the observation that both the mediobasal hypothalamus and the nucleus of the solitary tract in the caudomedial brainstem contain leucine-sensing cells that can modulate feeding behavior in response to leucine abundance (Figure 1A). Although these two apparently redundant sensing sites produce similar behaviors when injected with leucine, further studies are necessary to understand how they may differently be engaged in physiological contexts. Within each of these sensing sites, the population of leucine-sensing cells is heterogeneous. In the mediobasal hypothalamus, POMC neurons but also non-POMC neurons are activated in response to local leucine administration (26). Likewise, in the NTS, leucine-sensing cells are diverse and comprise POMC neurons, catecholaminergic neurons, and maybe as yet uncharacterized cell types (90). However, this is based on the sole use of c-FOS as a marker of neuronal activation, and we know very little about the electrophysiological response of these cells to leucine. Based on c-FOS studies, only a sub-population of cells within the MBH or NTS respond to leucine (26, 90), but what makes a cell leucine-sensitive or not is an open question, and whether leucine-sensing cells share a common molecular signature remains to be determined. To date, only arcuate POMC neurons have been shown to depolarize in response to leucine bath application in slice preparations, and the underlying mechanisms remains to be characterized (26, 92) (Figure 1B).

The detailed analysis of the feeding response to increased leucine levels within the hypothalamus or brainstem further supports the idea that leucine sensing engages multiple behavioral output circuits to control food intake. Indeed, meal pattern analyses show that the reduction in food intake measured in food-deprived rats and mice who received brain injections of leucine results from the alteration of various components of the feeding sequence: increased first meal latency (and therefore decreased orexigenic tone), decreased meal size and decreased meal frequency. One circuit implicated in the acute reduction in meal size following hypothalamic leucine sensing involves hypothalamic POMC neurons, their melanocortinergic projections to PVH oxytocin neurons and oxytocinergic projections to the nucleus of the solitary tract in the caudomedial brainstem (26). However, pharmacological inhibition of this circuit is neither sufficient to suppress the rapid reduction in first meal latency nor entirely blunt the anorectic response to leucine, suggesting that other circuits are involved in the overall behavioral response to brain leucine detection. One possible candidate to mediate the immediate increase in first meal latency following leucine hypothalamic administration in fasted rodents is AgRP neurons. This neuronal population is critical to the development of hunger and foraging during food deprivation (94, 95). Consistent with this possibility, leucine was found to regulate AgRP expression in hypothalamic GT1-7 cells (88). However, whether leucine can rapidly affect electrical or synaptic activity of AgRP neurons is unknown, and more generally, how changes in extracellular concentrations of leucine can rapidly and more chronically affect neuronal activity remains poorly characterized.

Activation of mTORC1 has been consistently associated with brain leucine sensing in the regulation of feeding (86). mTORC1 is an evolutionary-conserved signaling pathway that couples nutrient and growth factor sensing in the control of protein synthesis, growth, cell cycle progression and other processes (96). Within 30 min following discrete injections of leucine into the rodent MBH or NTS, phosphorylation of one of the major effector of mTORC1, p70 S6 kinase 1 (S6K1), increases in the respective site (26, 90). In rats, co-administration of rapamycin, a specific mTORC1 inhibitor, blunts leucine’s effect on meal size and early hypophagia, implicating activation of mTORC1 in the mechanisms underlying leucine-sensing in hypothalamic and brainstem feeding-regulating circuits. Further evidence in support for a role of mTOR in the regulation of feeding indicates that inhibition of MBH or Dorsal Vagal Complex mTOR signaling with rapamycin in fed rodents rapidly drives feeding and increases meal size (26, 86, 90). Conversely, constitutive activation of S6K1 inhibits feeding *via* a specific decrease in meal size in both the MBH and the Dorsal Vagal Complex. These data support a role for endogenous mTOR signaling in the control of meal size and foraging behavior. Interestingly, activation of neuronal TOR signaling has also been implicated in the control of feeding in drosophila. In this invertebrate, activation of p70 S6K1 signaling in neurons that control feeding produces hypophagia in fasted larvae, whereas its down-regulation produces increased foraging and feeding in fed larvae, and these responses engage neuropeptide Y-like signaling (97). Collectively, these data indicate that mTOR in discrete nutrient-sensing neurons is an evolutionary-conserved regulator of feeding behavior.

To better characterize the neurochemical populations of the MBH in which mTOR signaling is important in the control of feeding and metabolism in mice, Smith et al. generated mice with germline deletion of S6K1 from either POMC or AgRP neurons (92). Unexpectedly, a thorough metabolic phenotyping revealed that both lines had normal food intake, feeding behavior and energy expenditure under various experimental conditions. S6K1 deletion in both POMC and AgRP neurons reduced neuronal excitability, and reduces synaptic strength in AgRP neurons, resulting in impaired POMC and AgRP tone in these mice, but these alterations are not sufficient to produce a phenotype. This latter observation is consistent with reports showing that germline loss of AgRP neurons fail to affect energy balance, which supports the idea that feeding-regulatory circuits are plastic during development (98). This raises the possibility that S6K1 loss of function is compensated for in these lines. Further investigations are required to clarify the role of S6K1 in AgRP and POMC neurons in the regulation of energy balance in adult rodents.

As mentioned above, increased mTOR signaling is not sufficient to account for decreased meal frequency following MBH or NTS leucine administration (26, 90). We hypothesized that intracellular leucine metabolism, leading to ATP production, may contribute to neuronal leucine sensing in a mechanism analogous to the pancreatic and brain glucose sensing mechanism (99–101). Leucine undergoes intracellular metabolism, producing α-ketoisocaproic acid (KIC) and isovaleryl – CoA, respectively by branched-chain amino acid transferase (BCAT) and branched-chain ketoacid dehydrogenase (BCKDH), leading to the production of TCA cycle intermediates and ATP production that could contribute to leucine sensing in the MBH. Consistently, KIC injection into the mouse MBH suppressed feeding but through a specific decrease in meal number, without affecting meal size, suggesting that this mechanism is not recruited for the acute effects of leucine on foraging and meal size (26). Moreover, injection of an activator of BCKDK, resulting in an increase in the metabolism of leucine, caused a suppression of food intake, due to specific reduction in meal number. In addition, data obtained in mice bearing a whole body deletion of BCATm, expressed in astrocytes in the CNS and peripheral tissues, suggest that long-term high BCAA levels in the brain affect dietary preferences (102). This deletion produces high brain leucine concentrations. Although energy intake does not differ between the KO and the controls, these mice have a higher preference for a low BCAA diet over a normal diet, indicating that chronic abundance of BCAA in the brain induces changes in food choices that do not require BCAA transamination.

To our knowledge, leucine is the only EAA that has been found to produce an anorectic response when administered alone (Table 1). Orexin/hypocretin neurons, an orexigenic neuronal population in the lateral hypothalamus, have been shown to respond specifically to non-EAA using c-FOS staining and slice electrophysiology (103). The depolarization of these neurons in response to non-EAA was mediated by System A amino acid transporters and a suppression of the hyperpolarizing activity of K_ATP_ channels. Unlike in the MBH, leucine did not activate these neurons, as assessed by c-FOS immunohistochemistry and electrophysiology. The sensing of non-EAA was shown to be mTOR independent. Whether this sensing mechanism modulates feeding behavior remains to be established.

## Diets with Varying Protein Content: The Protein Leverage Hypothesis

As discussed above, a number of species avoid and develop conditioned aversion for diets very low in protein or with imbalanced EAA compositions. In contrast, marginally low-protein diets (LP, 8–10% of energy as protein in rats) with balanced EAA profiles induce a hyperphagic response restoring nitrogen and EAA intakes which, together with various metabolic adaptions, are sufficient to enable growth (87, 91, 104–108). Conversely, high-protein diets (HP, 20–70% of energy as protein) produce a sustained decrease in energy intake that is not caused by taste aversion even at very high-protein levels (109, 110). These bidirectional behavioral responses to shifts in dietary protein content are evolutionary conserved from insects to humans (111), and protein intake has been proposed to be regulated independently of energy intake (7, 112). In the following section, we will highlight the literature describing the role of brain amino acid sensing in the bidirectional regulation of energy intake in response to changes in dietary protein content.

### Response to Low-Protein Diets

Rats develop hyperphagia (starting day 2) following transition to a 10% protein diet (87, 113), but little is known about the behavioral components of this response, and to our knowledge, meal pattern analysis has not been performed in this context. Plasma levels of most EAA drop in the first 24 h after shift to a LP diet, but this response is only transient (87), and consistently, brain levels of amino acids, including BCAA and AAA, are not affected by LP feeding (12, 87). However, plasma and brain levels of BCAA and AAA fail to increase in response to food ingestion in rats adapted to a LP diet (87), suggesting that the inability to detect amino acids in the postprandial period could reduce satiety and contribute to hyperphagia. Consistent with this hypothesis, icv administration of leucine produces anorexia in LP-fed rats (87). To directly test the role of brain BCAA sensing in the hyperphagic response to LP diets, Morrison et al. measured energy intake in rats fed LP diets supplemented with leucine or BCAA (87). None of these treatments suppressed the hyperphagic response to LP diets, but confirmation that they produce increases in brain BCAA levels is missing. The authors went on and treated LP-fed rats with icv chronic amino acid infusions (87). They found that icv amino acid infusions could only partially blunt LP-induced hyperphagia, suggesting that the hyperphagic response to LP diets primarily involves peripheral amino acid sensing sites. These data argue against a role for direct brain amino acid detection in LP-induced hyperphagia but are not sufficient to rule out a contribution of brain amino acid sensing is this adaptation, as a lack of dynamic changes in BCAA or leucine levels in discrete nutrient-sensing regions, typically in the postprandial period, may contribute to reduced satiety in this model.

Data from the same group implicate hepatic FGF21 production in the metabolic and feeding responses to LP diets (91, 114). FGF21 circulating levels are dramatically increased in response to LP diets in mice, rats, and humans (91), and remarkably, *Fgf21* knockout mice are protected against the metabolic effects of LP diets (114). Using *Gcn2* knockout mice, the authors provided evidence suggesting that hepatic GCN2 responds to LP diets and promotes FGF21 production and release. These data clearly implicate FGF21 signaling in the metabolic adaptations to protein restriction, but whether brain amino acid sensing contributes to these responses remains to be clarified (Figure 1B). Interestingly, mTOR signaling interacts with GCN2 signaling (115, 116) and bidirectionally regulates FGF21 production (117), suggesting that multiple amino acid sensing pathways may orchestrate the overall metabolic and feeding responses to LP diets.

### Response to High-Protein Diets

At the other end of the spectrum, high-protein diets have been consistently shown to reduce energy intake in multiple species, from flies to humans (7, 111). When rats previously adapted to a normal-protein diet are offered a HP diet, they immediately decrease their food intake and progressively but incompletely re-increase food intake on the following days (118–121). Gut amino acid sensing and recruitment of local vagal afferents have been implicated in these responses, as reviewed in Ref. (11, 122). However, vagal afferents are not sufficient to produce the anorectic response to HP diets, as subdiaphragmatic vagotomy does not abolish the ability of a HP diet to reduce food intake (123). Brain detection of increased amino acid levels, particularly BCAA, may therefore also contribute.

Meal pattern analysis of rats exposed to a HP diet indicates that the initial important anorectic response to HP diets is mainly driven by a transient decrease in meal size, whereas after adaptation to the diet, decreased meal frequency is primarily accounting for the reduced energy intake (109). Ropelle et al. implicated activation of hypothalamic mTOR signaling in this response and found interestingly that although adaptation to a HP diet induced a chronic increase in brain leucine concentrations, hypothalamic mTOR signaling was only transiently increased (89). Thus, transient increase in hypothalamic mTOR signaling could account for the transient decrease in meal size during early exposure to HP feeding. In contrast, increase hypothalamic leucine catabolism may underlie the chronic decrease in meal frequency. However, there is currently no direct evidence to support this interpretation, and research in this field would significantly progress with the identification of a central mechanism specifically mediating the identification of protein abundance. Likewise, little is known about the neurocircuits involved in the hypophagic response to HP diets. Expression of *Pomc*-, *Npy*-, and *Agrp*-feeding neuropeptides is altered in response to a HP diet in rats (89), but these findings have been recently challenged (124), leaving open the contribution of melanocortinergic feeding circuits in the hypophagic response to increased dietary protein intake.

## Conclusion

**Figure 2:**
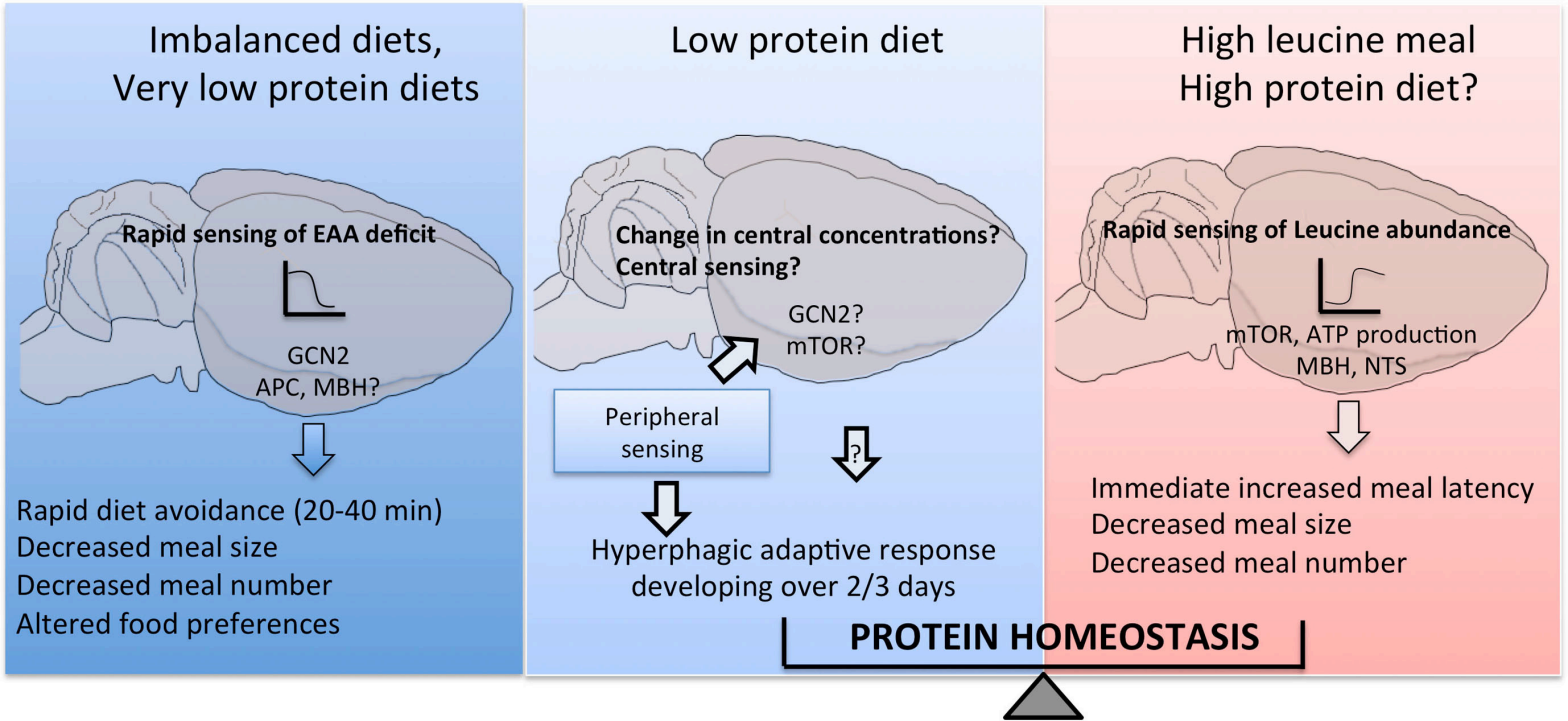
**Central detection of amino acid availability in the regulation of feeding behavior**. Discrete cell populations in the brain monitor extracellular amino acid availability through a variety of mechanisms and engage downstream feeding-regulatory circuits to prevent or limit the ingestion of diets with imbalanced essential amino acid content, produce hyperphagia if dietary protein content is low and conversely reduce food intake if dietary protein content is high.

Collectively, the data reviewed here support a role for a distributed network of discrete brain regions in primary amino acid sensing in the control of multiple behavioral responses to changes in dietary amino acid intake, as summarized in Figure 2. Many gaps remain to be filled to complete our understanding of these processes, and a key poorly described step is the intracellular coupling of intracellular amino acid availability to neuronal electrical and synaptic activity, as highlighted in Figure 1B. Does this coupling rely on specific intracellular components that could perhaps represent a unique molecular signature of amino acid sensing neurons, making these cells amenable to molecular genetics? Identifying a molecular marker for amino acid sensing neurons would prove extremely useful in the characterization of neurocircuits engaged downstream from primary brain sensors to regulate feeding behavior.

Brain amino acid sensing also modulates feeding behavior on a chronic basis and in some cases affects food preferences. The neurobiological substrates supporting these chronic changes are poorly characterized, and how dietary amino acids may affect the remodeling of central circuits regulating energy balance is unknown.

Central amino acid sensing has also been implicated in the regulation of metabolism and glucose homeostasis. Studies in flies and rodents suggest that decreased protein intake accounts for the beneficial metabolic effects of caloric restriction (3, 4, 125). Further work is required to determine how central amino acid sensing processes implicated in the control of feeding behavior may also coordinate metabolic effectors of energy balance and possibly mediate the beneficial effects of caloric restriction of metabolic health and lifespan.

Lastly, although we did not discuss in depth the evidence supporting the concept of protein homeostasis, understanding how distributed peripheral and central amino acid sensors monitor amino acid quantitative and qualitative availability to adjust feeding behavior is a long-term challenge in the field. The characterization of this “homeostatic” control could help identify novel targets for the prevention or treatment of obesity, potentially uncoupling the beneficial satiety effects of protein from their deleterious consequences on metabolic health.

## Author Contributions

Both authors contributed to the preparation of the manuscript.

## Conflict of Interest Statement

The authors declare that the research was conducted in the absence of any commercial or financial relationships that could be construed as a potential conflict of interest. The reviewer SF and handling Editor declared their shared affiliation, and the handling Editor states that the process nevertheless met the standards of a fair and objective review.

## Abbreviations

eIF2, eukaryotic initiation factor 2α; AAA, aromatic amino acids; AgRP, agouti-related peptide; APC, anterior piriform cortex; ATP, adenosine triphosphate; BBB, blood–brain barrier; BCAA, branched-chain amino acids; BCAT, branched-chain amino acid transferase; BCKDH, branched-chain ketoacid dehydrogenase; CNS, central nervous system; EAA, essential amino acids; GABA, γ-aminobutyric acid; GCN2, general control non-derepressible 2; HP, high protein; icv, intracerebroventricular; KIC, α-ketoisocaproic acid; KO, knock out; LP, low protein; MBH, mediobasal hypothalamus; mTOR, mammalian target of rapamycin; NPY, neuropeptide Y; NTS, nucleus tractus solitarii; OVN, overnight; POMC, pro-opio melanocortin; PVH, paraventricular nucleus of the hypothalamus; TCA, tricarboxylic acid; WT, wild type.
